# A CRISPR/Cas12a-assisted rapid detection platform by biosensing the *apxIVA* of *Actinobacillus pleuropneumoniae*

**DOI:** 10.3389/fmicb.2022.928307

**Published:** 2022-09-09

**Authors:** Tian Luan, Lu Wang, Jiyu Zhao, Hui Luan, Yueling Zhang, Chunlai Wang, Paul R. Langford, Siguo Liu, Wanjiang Zhang, Gang Li

**Affiliations:** ^1^State Key Laboratory of Veterinary Biotechnology, Division of Bacterial Diseases, Harbin Veterinary Research Institute, Chinese Academy of Agricultural Sciences, Harbin, China; ^2^College of Veterinary Medicine, Xinjiang Agricultural University, Xinjiang, China; ^3^College of Veterinary Medicine, Northeast Agricultural University, Harbin, China; ^4^Section of Paediatric Infectious Disease, Department of Infectious Disease, Imperial College London, St. Mary’s Campus, London, United Kingdom

**Keywords:** CRISPR, Cas12a, crRNA, *Actinobacillus pleuropneumoniae*, diagnostics

## Abstract

*Actinobacillus pleuropneumoniae* is an important respiratory pig pathogen that causes substantial losses in the worldwide swine industry. Chronic or subclinical infection with no apparent clinical symptoms poses a challenge for preventing transmission between herds. Rapid diagnostics is important for the control of epidemic diseases. In this study, we formulated an *A. pleuropneumoniae* species-specific *apxIVA*-based CRISPR/Cas12a-assisted rapid detection platform (Card) that combines recombinase polymerase amplification (RPA) of target DNA and subsequent Cas12a ssDNase activation. Card has a detection limit of 10 CFUs of *A. pleuropneumoniae*, and there is no cross-reactivity with other common swine pathogens. The detection process can be completed in 1 h, and there was 100% agreement between the conventional *apxIVA*-based PCR and Card in detecting *A. pleuropneumoniae* in lung samples. Microplate fluorescence readout enables high-throughput use in diagnostic laboratories, and naked eye and lateral flow test readouts enable use at the point of care. We conclude that Card is a versatile, rapid, accurate molecular diagnostic platform suitable for use in both laboratory and low-resource settings.

## Introductions

The bacterium *Actinobacillus pleuropneumoniae* is a member of the *Pasteurellaceae* family and is the cause of porcine pleuropneumonia, which is characterized by hemorrhagic, fibrinous, and necrotic lung lesions (Frey, 1995). *A. pleuropneumoniae* is responsible for substantial morbidity, mortality, and economic losses in the worldwide swine industry (Losinger, 2005). Nineteen serovars have been identified based on capsule loci (Stringer et al., 2021a), and the isolates are assigned as biovar 1 and 2 if they are NAD-dependent or -independent, respectively (Serrano-Rubio et al., 2008). Vaccination is considered the best approach to control epidemic diseases, but there are currently no commercially available vaccines that provide cross-protection against all serovars (Loera-Muro and Angulo, 2018).

Therefore, to prevent and control the epidemic of porcine pleuropneumonia, rapid diagnostics play a vital role (Stringer et al., 2021b). Serology, e.g., ApxI-IV ELISAs, can be used to monitor the presence of clinical infections (Nielsen et al., 2000; Andresen et al., 2002; Wei et al., 2012; Gimenez-Lirola et al., 2014), but it is less suitable for the detection of carrier pigs in tonsils or nasal cavities (Sidibe et al., 1993; Chiers et al., 2002). Typically, the identification of *A. pleuropneumoniae* is established by selective bacterial examination followed by phenotypic and/or molecular tests, e.g., based on the presence of the species-specific *apxIVA* gene (Schaller et al., 2001). This can take days to get the results back. So, there is a need for more a rapid, especially point-of-care, testing to enable early antibiotic treatment and/or changes in herd management to control disease (Stringer et al., 2021b). Now, there has been a move toward molecular diagnostics, such as PCR (Gram et al., 1996; Klein et al., 2003; Hricinova et al., 2010) and quantitative real-time PCR (qPCR) (Tobias et al., 2012; Watt et al., 2020). These techniques are well developed and are typically based on the *apxIVA* gene (Schaller et al., 2001). However, these methods still have some limitations. For example, they require expensive thermal cycling instruments and skilled personnel, which have limited their application at the point of care and in low-resource settings. Recently, isothermal amplification techniques, such as recombinase polymerase amplification (RPA) (Li et al., 2019) and loop-mediated isothermal amplification (LAMP) (Yang et al., 2009), have been used to detect *A. pleuropneumoniae* and have potential use for rapid point-of-care diagnostics. Here, we describe a rapid *A. pleuropneumoniae* detection platform based on a combination of RPA with elements of the adaptive clustered regularly interspaced short palindromic repeats (CRISPR)/Cas immune system.

Bacteria and archaea have evolved CRISPR and their associated proteins (Cas), collectively referred to as the CRISPR-Cas immune system, to specifically target exogenous viral infections (Koonin and Makarova, 2019). The CRISPR-Cas systems have precise double-stranded DNA (dsDNA) cleavage activity due to their nucleotide sequence-specific recognition capabilities (Zetsche et al., 2015). The CRISPR-Cas9 system is widely used for genome editing in various organisms, such as bacteria, plants, and animals (Jiang et al., 2015; Burgio, 2018; Lu and Tian, 2022). However, recently discovered Cas12a and Cas13a systems have features that are different from the Cas9 system (Zetsche et al., 2015). For example, they can be complexed with a CRISPR RNA (crRNA) containing a programmable spacer sequence to form a nuclease-inactive ribonucleoprotein (RNP) complex. When the RNP binds to complementary target DNA/RNA, their collateral cleavage activity is activated and then precisely cleaves the surrounding single-stranded DNA (ssDNA) (Chen et al., 2018) or RNA (ssRNA) substrates (Fozouni et al., 2021). The substrate can be linked by a fluorophore-quencher pair, and the cleavage activity is detected by measuring the fluorescence (East-Seletsky et al., 2016). This has permitted the development of nucleic acid detection platforms for different pathogens that combine the CRISPR-Cas12a and Cas13a systems and gene amplification, such as DETECTR (Chen et al., 2018), SHERLOCK (Gootenberg et al., 2018), and HOLMES (Li et al., 2018), which have high sensitivities (at the single-molecule level) and specificities (single base resolution). CRISPR/Cas-based diagnostic technology had been successfully applied to detect many common pathogens, e.g., *Mycobacterium tuberculosis* (Xiao et al., 2020), African swine fever virus (ASFV) (Lu et al., 2020), SARS-CoV-2 (Cao et al., 2021), Zika virus (ZIKV), Dengue virus (DENV) (Gootenberg et al., 2018), and human papillomavirus (HPV) (Chen et al., 2018). The Cas13-based SHERLOCK system requires the target DNA to be reverse-transcribed to RNA for detection by Cas13a (Gootenberg et al., 2018). For bacterial genome targets, the use of the DNA-sensing Cas12 avoids the need for transcribing RNA to DNA (Chen et al., 2018). Therefore, the Cas12a-based system using a single-stranded DNA probe is more suitable for the detection of bacterial pathogens.

To our knowledge, no CRISPR-Cas12a-based platform has been developed to detect *A. pleuropneumoniae*. Here, we report the development of a rapid CRISPR-Cas12a-based platform for direct detection of *A. pleuropneumoniae* based on the species-specific gene *apxIVA*, which we have named the Cas12a/crRNA-Assisted Rapid Detection (Card) platform.

## Materials and methods

### Design and preparation of crRNA

Since the *apxIVA* gene was reported to be a suitable locus for the identification of *A. pleuropneumoniae* (Schaller et al., 2001; da Costa et al., 2004; Tobias et al., 2012), we chose it as the target sequence for the design of crRNA probes. Eight species-specific crRNA probes were designed based on a conserved region of the *apxIVA* gene (Park et al., 2015). The DNA template for crRNA transcription was synthesized by Comate Biotech with a T7 promoter sequence added at its 3′ end. *In vitro* transcription was performed using the HiScribe T7 Quick High Yield RNA Synthesis kit (New England Biolabs, United States) following the manufacturer’s recommendations. The final translation products were treated with the addition of DNase I to remove template DNA and subsequently purified using the RNA Clean and Concentrator kit (Zymo Research, United States) following the manufacturer’s protocol. crRNA concentration was quantified in a Nanodrop spectrophotometer (IMPLEN, United States). The sequences of all the oligonucleotides synthesized (Comate Biotech, China) in this study are shown in Table 1.

**TABLE 1:** The crRNAs and primers used for amplification.

Name	Sequences (5′-3′)
crRNA1	UCCGAACUUUGGUUUAGCCG
crRNA2	GUUUAGCCGAGAAAAUAACG
crRNA3	GCCGAGAAAAUAACGAUUUG
crRNA4	AUAAUCAAAUCGUUAUUUUC
crRNA5	AUUAUUAAAUCAUUAUUAAG
crRNA6	UCCUCACUUAAUAAUGAUUU
crRNA7	AACCGUGACUUUAUCCUCAC
crRNA8	GAACCGUGACUUUAUCCUCA
RPA-up	ATATTAATTTATCCGAACTTTGGTTTAGCCGAGA
RPA-down	CGACTCAACCATCTTCTCCACCTGAGTGCTCAC
ssDNA	FAM-TTATT-TAMRA/FAM-TTATT-Biotin
P1	GCTTGCATGC**CTGCAG**TGGCACTGACGGTGAT
P2	CTGAATTCGAGCTC**GGTACC**GGCCATCGACTCAACCAT
ApxIVA-F	TGGCACTGACGGTGATGA
ApxIVA-R	GGCCATCGACTCAACCAT
ApxIVA-q-F	GGGGACGTAACTCGGTGATT
ApxIVA-q-R	GCTCACCAACGTTTGCTCAT
ApxIVA(probe)	FAM-CGGTGCGGACACCTATATCT-BHQ1

Underlined sequences are homologous with pUT18, and bold characters indicate the PstI (P1) and KpnI (P2) restriction endonuclease sites.

### Genomic DNA preparation from pathogen and clinical samples

Genomic DNA from *A. pleuropneumoniae* of different serovar reference strains and other common bacterial pathogens used in the specificity assay were extracted with the Genomic DNA Extraction kit (TIANGEN, China). Viral genomes were extracted with the AxyPrep Body Fluid Viral DNA/RNA Miniprep kit (Axygen Scientific, United States). The DNA from lung samples was extracted with the Tissue and Blood DNA extraction kit (TIANGEN, China) according to the manufacturer’s instructions.

### Recombinase polymerase amplification

The RPA primer sequences for *apxIVA* were designed by the Primer 5.0 software by setting the product length to 100–200 bp, with a size preference of 30–35 nt, and with a GC content between 30 and 70%. Standard RPA reactions were performed according to the manufacturer’s instructions of the TwistAmp Basic kit (TwistDx Ltd, United Kingdom) with minor modifications. Every single reaction contained 14.75 μL of rehydration buffer, 0.48 μM forward and reverse primers, an appropriate amount of genomic DNA, 1.25 μL of magnesium acetate (MgAc), and sterile water up to 20 μL. The mixture was incubated at 37°C for 15 min.

### Reaction assay

LbCas12a-crRNA complexes were preassembled by incubating 1 μM LbCas12a (New England Biolabs, United States) and 1.25 μM crRNA at room temperature for 10 min. DNA targets of various concentrations were dissolved in 1 × Binding Buffer (10 mM Tris-HCl, pH 7.5, 50 mM NaCl, 5 mM MgCl_2_, 0.1 mg/mL bovine serum albumin [BSA]) and mixed with LbCas12a-crRNA complexes and 500 nM ssDNA reporter probe in 20 μL reaction volume. In the assay, the final concentrations of LbCas12a and crRNA were 50 and 62.5 nM, respectively. The mixture was incubated in a constant temperature incubator at 37°C for 15 min.

### Readout formats

The detection mechanism is based on the non-specific ssDNA probe cleavage by Cas12a-crRNA complexes. To report the presence of *A. pleuropneumoniae* genomic DNA from different samples, ssDNA probes with different label methods were used. Three different readouts were used. An ssDNA probe (5′-FAM-TTATT-TAMRA-3′) linked with fluorescent and quencher groups was used for fluorescence-based detection either by the naked eye under blue light (Sangon Biotech, China), or by a fluorescent microplate reader (PerkinElmer EnSpire, United States). An ssDNA probe (5′-FAM-TTATT-Biotin-3′) with fluorescein on one end and biotin on the other was used for detection colorimetrically in lateral flow strips (Milenia Biotec GmbH, United Kingdom). On the lateral flow strips, the antibodies labeled with gold nanoparticles (NPs) were embedded in the sample area of the strip and could bind the ssDNA reporter to form a dark purple color line. When no genomic DNA of *A. pleuropneumoniae* appears in the reaction system, all the uncleaved ssDNA reporter would be captured by the streptavidin present in the control line, and only one purple line on the strip would be present. In contrast, a positive result arises from the reporter being cleaved and the gold NP–labeled antibodies continuously flowing toward the test line covered with anti-FAM antibodies and forming another dark purple color line, which indicates the presence of the target in the system. All the colorimetric processes were completed in less than 5 min.

### Specificity of card

To evaluate the specificity of this detection platform, apart from the *A. pleuropneumoniae* serovar 7 strain S8 (Li et al., 2012), several other common pathogens and closely related species were also tested. The *Glaesserella parasuis* serovar 5 reference strain Nagasaki (Frandoloso et al., 2012) and *Streptococcus suis* serovar 2 strain 05ZYH33 (Feng et al., 2014) were stored in our laboratory. The *Pasteurella multocida* CVCC438 and *Salmonella enterica* serovar *choleraesuis* CVCC 79500 were purchased from the China Veterinary Culture Collection Center (CVCC). The genome of porcine circovirus type 2, porcine pseudorabies virus, and porcine epidemic diarrhea virus were kindly provided by other research groups of the Harbin Veterinary Research Institute, Chinese Academy of Agricultural Sciences, China. The specificity of the Card platform for these pathogens was assessed by all the readout formats. Additionally, the species from the *Pasteurellaceae* family (*Actinobacillus suis*, *Actinobacillus equuli*, *Actinobacillus lignieresii*, *Actinobacillus indolicus*, Actinobacillus porcitonsillarum, *Actinobacillus porcinus*, *Actinobacillus pneumotropica*, and *Actinobacillus minor*) and all the reference strains (serovar 1, strain 4074; serovar 2, S1536; serovar 3, S1421; serovar 4, M62; serovar 5a, K17; serovar 6, Fem∅; serovar 7, WF83; serovar 8, 405; serovar 9, CVJ13261; serovar 10, D13039; serovar 11, strain 56153; serovar 12, strain 1096; serovar 13, strain N273; serovar 14, strain 3906; serovar 15, strain HS143; serovar 16, strain A-85/14; serovar 17, strain 16287-1; serovar 18, strain 7311555; serovar 19, strain 7213384-1) were also tested. Another 10 field isolates [serovar 1 (isolates 2 and 5), serovar 2 (isolates 1, 3, and 8), serovar 7 (isolates 4, 6, 7, 9, and 10)], which had been isolated from the lungs of suspected *A. pleuropneumoniae* infections in different farms of China and identified by PCR (Stringer et al., 2021a), were also tested. The specificity for these species was assessed in a fluorescent microplate reader.

### Sensitivity determination of CRISPR-Cas12a using a conserved cloned *apxIVA* fragment

To evaluate the sensitivity of the Card platform, we first determined the sensitivity using serial dilutions of the plasmid template. The fragment containing the chosen conserved region of *apxIVA* was amplified by PCR with primer pairs P1 and P2. Then, the PCR products were purified by using a gel extraction kit following the manufacturer’s protocol. The final purified fragment was ligated to the pUT18 plasmid (Euromedex, United States). The plasmid concentration was then quantified by Nanodrop spectrophotometry, and the initial copy number was calculated according to plasmid length and concentration. The serially diluted plasmid was used as a template in the RPA reaction system.

Additionally, serial dilutions of pure culture of an *A. pleuropneumoniae* strain were also used to determine the sensitivity. The *A. pleuropneumoniae* serovar 7 strain S8 was cultured overnight, serial dilutions were plated on TSA medium, and the colony forming units (CFUs) were counted. Subsequently, 1 mL cultures of different dilutions (10^6^ to 10^0^ CFUs/mL) were centrifuged at 5,000 × *g* for 5 min, resuspended in 100 μL PBS buffer, boiled for 10 min, and samples were centrifuged at 12,000 × *g* for 10 min. The resulting supernatants were used as templates in the RPA reaction system. All the tests were done in triplicate. Meanwhile, to compare the LOD of the Card platform, the same template was also subjected to the *apxIVA*-based PCR (Schaller et al., 2001) and qPCR (Tobias et al., 2012). The qPCR analysis was done using an Applied Biosystems QuantStudio 5 thermocycler with primer pair *ApxIVA*-q-F and *ApxIVA*-q-R.

### Assay validation with lung samples

To further validate utility, genomic DNA isolated from pig lung tissues was tested in the Card platform. These lung samples were obtained from experimental infection studies in pigs challenged with the *A. pleuropneumoniae* serovar 7 strain S8 that had been carried out in our laboratory previously (Xie et al., 2017). The lung tissues (20–50 mg) were mixed with PBS buffer, homogenized for 10 min, and centrifuged at 3,000 × *g* for 5 min, and a 50-μL supernatant was spread on the TSA medium at 37°C for 12 h. Suspected *A. pleuropneumoniae* colonies were identified by PCR with the primer pair *ApxIV*-F and *ApxIV*-R. Genomic DNA from homogenized lung tissues was extracted according to the manufacturer’s instructions. Confirmation of *A. pleuropneumoniae* for genomic DNA was carried out by PCR with the same primer pair. The CFUs from these samples were also assessed by qPCR.

### Statistical analysis

The GraphPad Prism v.8.1.2 software was used for data analysis and graphic design. The data in the figures were expressed as mean ± standard deviation.

## Results and discussion

### Overview of the Cas12a/crRNA-assisted rapid detection (card) platform

**FIGURE 1:**
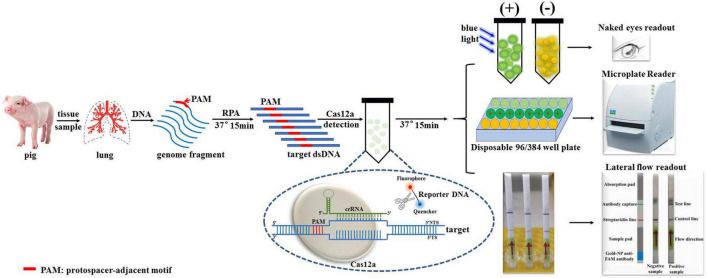
Schematic diagram of Cas12a/crRNA-assisted rapid detection (Card) platform for identification of *A. pleuropneumoniae*. Gene targets are specifically pre-amplified from genome fragments isolated from clinical samples by RPA. The specific crRNA probes were designed to target amplified fragments, and mixed with Cas12a/crRNA/ssDNA-FQ. Once the target DNA is recognized by the complex formed by Cas12a and crRNA, Cas12a exhibits non-specific endonuclease activity and cleaves the fluorescently labeled reporter (fluorophore [F] is quenched by a quencher [Q]) resulting in fluorescence, which is detected in a microplate reader, or observed by the naked eye under blue light.

A schematic overview of the Card platform for the identification of *A. pleuropneumoniae* is shown in Figure 1. First, the nucleic acid is extracted from the clinical sample by a commercial genome extraction kit, and the target DNA is amplified by RPA for 15 min. The amplified product is mixed with crRNA, Cas12a, and a quenched fluorescence reporter, ssDNA (total time = 15 min). When the amplified target DNA is recognized by the specific crRNA probe, Cas12a is activated, and the complex cleaves the free ssDNA reporter in the reaction mixture. Fluorescence can be directly detected in an appropriate microplate reader or colorimetrically in lateral flow strips or tubes. The entire detection process can be completed within 1 h.

### Screening of the optimal crRNA

**FIGURE 2:**
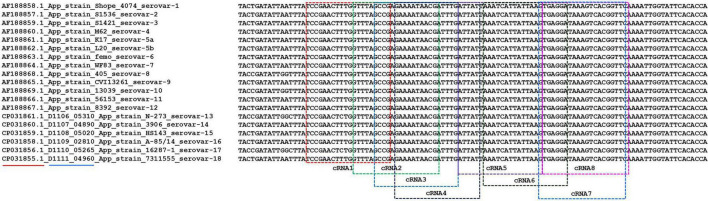
The alignment of partial coding sequence of *apxIVA* of *A. pleuropneumoniae* serovars 1–18 downloaded from the NCBI database. Red underline indicates the accession number of the sequence in the NCBI database. Blue underline indicates the locus tag of the gene in the genomic sequence. The different colored frames indicate the recognized region of different crRNAs.

**FIGURE 3:**
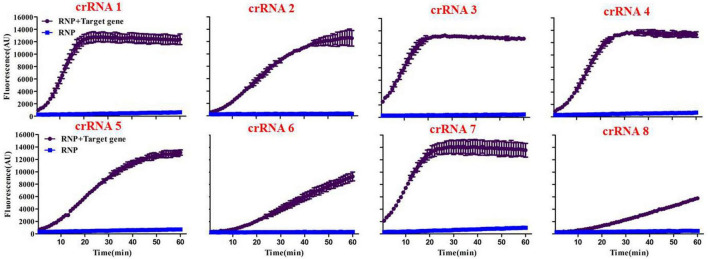
Screening of specific crRNAs by the Card platform. The performance of crRNA 1 to crRNA 8 was determined by testing 50 nM of each RNP individually against 10^5^ copies/μL of plasmid including a conserved region of the *A. pleuropneumoniae*-specific *apxIVA* gene. RNP = no target DNA in the reaction system. Data are represented as mean ± standard deviation.

Initially, we designed eight crRNA probes (Table 1) based on the species-specific *apxIVA* gene of *A. pleuropneumoniae* (Schaller et al., 2001). Every Cas12a-crRNA RNP recognized a single 20-nucleotide region of the *apxIVA* gene (Figure 2), and each crRNA was initially individually tested in a microplate detection assay. The assay used LbCas12a and a quenched fluorescence reporter, ssDNA, together with *in vitro* transcribed crRNA, which had been designed to correspond to a conserved region within the *apxIVA* gene. Initial testing was carried out with plasmid pUT18, which harbored a conserved region of the *apxIVA* gene. At a target DNA concentration of 1.0 × 10^5^ copies/μL, eight crRNAs with reactivity above the RNP alone control (RNP and probe but no target DNA) were identified. Similar trends in specific crRNA performance were found (Figure 3). For further studies, crRNA 7 was selected since it generated the highest fluorescence value in the shortest reaction time.

### Specificity of card

**FIGURE 4:**
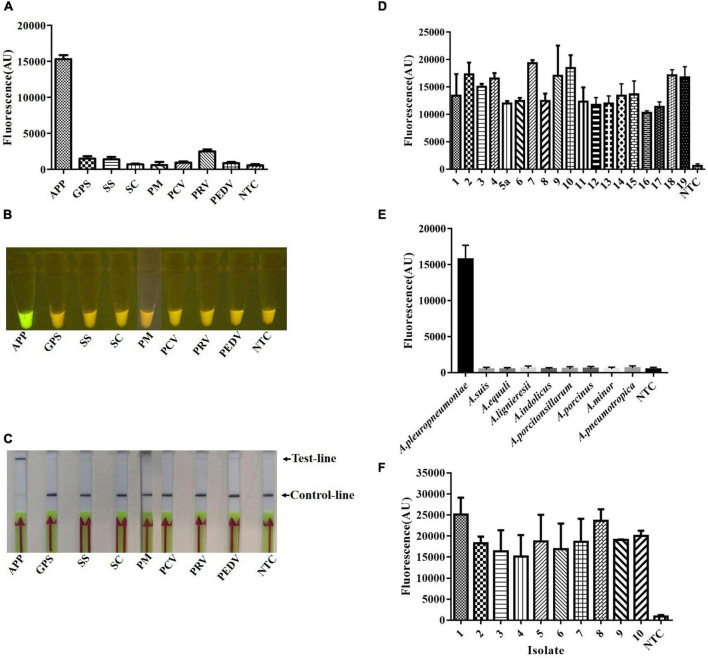
Specificity of the crRNA 7 probe for *A. pleuropneumoniae*, and detection in multiple formats. The specificity of Card was determined by using genomic or reverse-transcribed DNA extracted from the different pathogens associated with diseases in pigs. **(A)** Endpoint fluorescence of RPA amplification products detected by a fluorescence microplate reader. **(B)** Visual detection of RPA amplification products under blue light: *A. pleuropneumoniae* appears green and negative samples appear yellow. **(C)** Detection of *A. pleuropneumoniae* RPA amplification products in a lateral flow strip format. A purple control line is present in all lateral flow strips, but an additional line is present when *A. pleuropneumoniae* genomic DNA was used as the template. **(D)** Endpoint fluorescence (microplate reader) for genomic DNA from *A. pleuropneumoniae* serovars 1–19. **(E)** Endpoint fluorescence (microplate reader) for genomic DNA from related species to *A. pleuropneumoniae*. **(F)** Endpoint fluorescence (microplate reader) for genomic DNA from ten field isolates of *A. pleuropneumoniae* serovar 1 (isolate 2, 5), serovar 2 (isolate 1, 3, 8), serovar 7 (isolates 4, 6, 7, 9, and 10). In all cases, 50 ng genomic DNA or cDNA were used in the reaction system, and three replicates were conducted for each test, with one representative being shown. *A. pleuropneumoniae* (APP), *Glaesserella parasuis* (GPS), *Streptococcus suis* (SS), *Salmonella enterica subsp. enterica serovar choleraesuis* (SC), *Pasteurella multocida* (PM), Porcine circovirus (PCV), Pseudorabies virus (PRV), and Porcine Epidemic Diarrhea Virus (PEDV), non-template control (NTC). Error bars represent the standard deviations of the mean of the three replicates (*n* = 3).

A major advantage of CRISPR diagnostics is their high specificity. To confirm the specificity of the crRNA 7, we tested it against other pathogens, including *G. parasuis*, *Streptococcus suis*, *S. choleraesuis, P. multocida*, porcine circovirus, pseudorabies virus, and porcine epidemic diarrhea virus, which are common pathogens that infect pigs and cause severe economic losses. The specificity of the crRNA 7 probe was evaluated preliminarily using extracted genomic or reverse-transcribed DNA from these pathogens as appropriate. High-emitted fluorescence was found when using a microplate reader when the genome of *A. pleuropneumoniae* was used as a template, and there was no cross-reaction with the other pathogens evaluated (Figure 4A). Similarly, green fluorescence was observable under blue light for *A. pleuropneumoniae* but not for other pathogens (Figure 4B). In the lateral flow format, there was a clear purple control line with all samples but an additional purple line with *A. pleuropneumoniae* (Figure 4C). Meanwhile, the specificity for genomic DNA from pure cultures of reference serovars, closely related species, and clinical isolates was also evaluated. There was high fluorescence in the microplate format from all of the *A. pleuropneumoniae* serovar reference strains (Figure 4D), related species (Figure 4E), and the isolates with various serovars (Figure 4F). These results indicated that the crRNA 7 probe had high specificity for *A. pleuropneumoniae* and could be applied to the Card platform for use with multiple readout formats.

### Sensitivity of card

**FIGURE 5:**
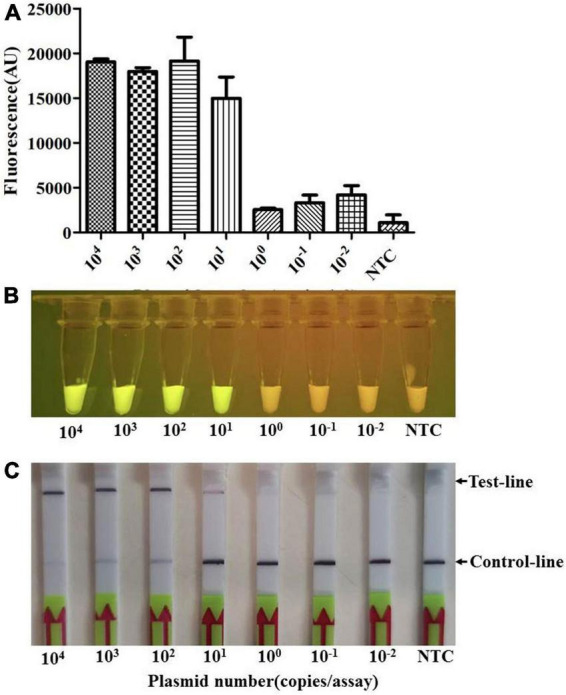
The limit of detection (LOD) of the Card platform for the plasmid sample. The LOD for the plasmid sample was determined by using the diluted plasmid containing *apxIVA* DNA insert as a template. **(A)** Endpoint fluorescence of RPA amplification products detected in a fluorescence microplate reader. Error bars represent the standard deviations of the mean of three replicates (*n* = 3). **(B)** Visual detection of RPA amplification products under blue light: positive samples appear green and negative samples appear yellow. **(C)** Detection of RPA amplification products in a lateral flow strip format. A purple control line is present in all lateral flow strips, but an additional line is present when the plasmid DNA was detected by the Card platform. NTC, non-template control. In all cases, three replicates were conducted for each test, with one representative being shown.

To determine the sensitivity of the Card platform, we first carried out serial dilutions of the plasmid template to determine the limit of detection for this platform. The plasmid DNA was prepared by ligated amplified *apxIVA* gene fragment to pUT18 vector, and the extracted plasmid was 10-fold diluted from a starting concentration of 10^9^ copies/μL. The results showed that, if more than 10^1^ copies (≈1 fg) of the plasmid are present in the reaction system, it can be successfully detected as positive (Figures 5A–C).

**FIGURE 6:**
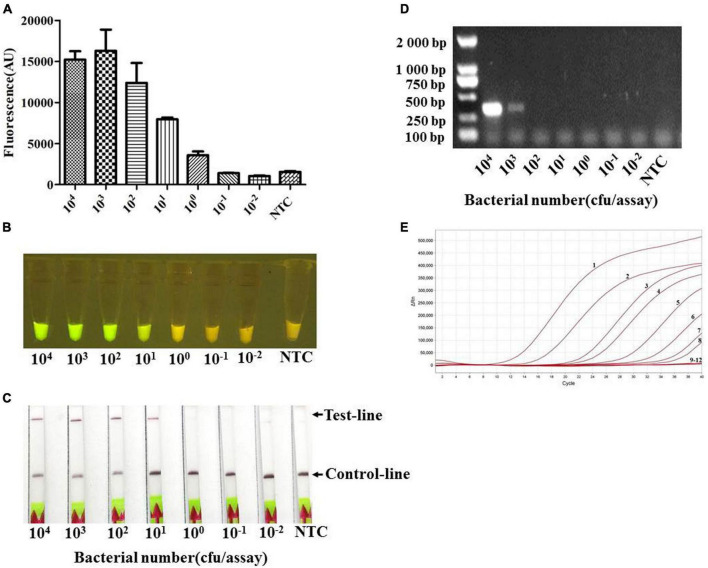
The limit of detection (LOD) of the Card platform for a pure culture of App S8 serovar 7. The LOD for different CFUs was determined by using the genomic DNA from serial dilutions of pure culture as a template. **(A)** Endpoint fluorescence of RPA amplification products detected in a fluorescence microplate reader. Error bars represent the standard deviations of the mean of the three replicates (*n* = 3). **(B)** Visual detection of RPA amplification products under blue light: positive samples appear green and negative samples appear yellow. **(C)** Detection of RPA amplification products in a lateral flow strip format. A purple control line is present in all lateral flow strips, but an additional line is present when the genomic DNA was detected by the Card platform. **(D)** The genomic DNA from serial dilutions of pure culture was detected by the PCR. **(E)** The genomic DNA from serial dilutions of pure culture was detected by quantitative real-time PCR. The numbers 1–8 represent the CFUs from 10^8^ to 10^1^ per assay, numbers 9–12 represent the CFUs from 10^0^ to 10^–2^, and NTC (non-template control) per assay.

In addition, the genomic DNA of serial dilutions of pure culture from *A. pleuropneumoniae* serovar 7 strain S8 in PBS buffer, equivalent to DNA from 10^4^ to 10^–2^ CFUs per reaction, was evaluated in the reaction system. The results indicated that the analytical sensitivity could reach 10^1^ CFUs (≈100 fg) per reaction (Figures 6A–C). This is consistent with qPCR (Figure 6E) and 100 times higher than PCR, where a positive band was only found when more than 10^3^ CFUs were included in the reaction system (Figure 6D).

### Application of card platform to lung samples

We examined whether the Card Platform could be used for the detection of *A. pleuropneumoniae* in lung samples. The genomic DNA from these samples was identified by PCR amplification of the *apxIVA* gene. The homogenized lung tissues were also spread on a selective medium and identified by colony PCR. A total of 30 positive and 5 negative samples were confirmed by both PCR methods. The bacterial load of positive samples was determined by qPCR with the genomic DNA from positive samples, and the results showed that the CFUs of positive samples ranged from 4.8 × 10^3^ to 3.6 × 10^6^ CFUs/g. All the genomic DNA were examined by the Card platform and observed with the naked eyes. The results showed that all *apxIVA* PCR positive samples produced green fluorescence, while all negative samples emitted yellow light. There was a 100% correlation between the Card platform and the *apxIVA* PCR method.

## Discussion

There is a need for rapid on-site diagnostics that can be used to detect the major pig pathogen *A. pleuropneumoniae*. In the present study, we combined RPA with CRISPR-Cas12a to formulate the Card platform to detect *A. pleuropneumoniae*. Initially, eight specific crRNAs were designed for binding to a conversed region of the *A. pleuropneumoniae* specific *apxIVA* gene. Although the aligned sequences were not from all of the 19 known *A. pleuropneumoniae* serovars, the region is high conserved between the *A. pleuropneumoniae* 1–18 reference strains. These designed crRNAs showed different specificity in target dsDNA recognition, and we chose the crRNA 7 to take forward as it showed the highest fluorescence. There was no cross-reaction with nucleic acid samples extracted from other common bacterial or viral pathogens of pigs. Such specificity is important for the successful application of such tests to the field (Alon et al., 2021; Gong et al., 2021; Liu et al., 2021; Ma et al., 2021).

To validate the sensitivity of the Card platform, DNA samples from different origins were used in the reaction system. The LOD of the Card platform could reach 10 copies of plasmid or CFUs of *A. pleuropneumoniae*. It was similar to that of qPCR (Tobias et al., 2012) and more sensitive than PCR, where the LOD was 10^3^ copies. When Card was used on lung samples, there was a 100% correlation with a conventional *apxIVA*-based PCR. This means that it had good agreement with the most widely used methods (Schaller et al., 2001; Fittipaldi et al., 2003; Klein et al., 2003). Due to the unavailability of samples, we did not assess whether Card can be used on the nasal or tonsillar material, which will be the subject of a future study. We speculate that, being a nucleic-based test, it will be effective for use on such samples, but its clinical utility remains to be determined. Now, all the tests were done using the genomic DNA extracted from pure cultures and lung tissues in a commercial kit. There were some simplified extraction protocols for sample preparation that had been developed to minimize the number of steps in the assay (Myhrvold et al., 2018; Joung et al., 2020), which would bring greater convenience to on-site detection.

The versatile readout styles of the Card platform make it not only suitable for high-throughput professional veterinary diagnostic laboratories (microplate reader format) but also for on-site point-of-care (naked eyes and lateral flow formats) diagnosis of *A. pleuropneumoniae*. In particular, Card is rapid (results in less than 1 h), which facilitates its use at the point of care, and does not require the use of more expensive instruments that are required for conventional PCR and qPCR. However, the reagent cost per test of the Card platform is higher than the commonly used PCR and qPCR (Rauch et al., 2021), two of the least expensive testing modalities currently available. This bottleneck may be gradually solved with the increasing popularity.

We conclude that the Card platform shows great promise as a rapid, point-of-care diagnostic for *A. pleuropneumoniae*.

## Data availability statement

The raw data supporting the conclusions of this article will be made available by the authors, without undue reservation.

## Author contributions

WZ and GL designed the experiments. TL, LW, JZ, WZ, and GL wrote the manuscript. TL, LW, and JZ performed the experiments. TL, LW, JZ, HL, YZ, and CW collected and processed materials. TL, LW, JZ, HL, and YZ analyzed the data. PL and SL revised the manuscript. All authors read and approved the submitted version.
